# Use of rivaroxaban in Germany: a database drug utilization study of a drug started in hospital

**DOI:** 10.1007/s00228-014-1697-7

**Published:** 2014-05-27

**Authors:** Kathrin Jobski, Dirk Enders, Ute Amann, Kiliana Suzart, Mari-Ann Wallander, Tania Schink, Edeltraut Garbe

**Affiliations:** 1 Leibniz Institute for Prevention Research and Epidemiology–BIPS GmbH, Achter Str. 30, 28359 Bremen, Germany; 2 MONICA/KORA Myocardial Infarction Registry, Central Hospital of Augsburg, Augsburg, Germany; 3 Helmholtz Zentrum München, German Research Center for Environmental Health (GmbH), Institute of Epidemiology II, Neuherberg, Germany; 4 Global Development–Global Epidemiology, Bayer Pharma AG, Berlin, Germany; 5 Department of Public Health and Caring Science Uppsala University, Uppsala, Sweden; 6 University of Bremen, Bremen, Germany

**Keywords:** Rivaroxaban, Drug utilization, Inpatient drug use, German Pharmacoepidemiological Research Database

## Abstract

**Purpose:**

The purpose of this drug utilization study was to describe the use of rivaroxaban in Germany during a time period in which approval was limited to the prevention of venous thromboembolism following hip or knee replacement. Additionally, we explored the feasibility of reconstructing inpatient drug use of rivaroxaban in a database where with a few exceptions inpatient prescribing information is not available.

**Methods:**

Source of data was one statutory health insurance providing data on about seven million insurants throughout Germany. Analyses were based on a cohort of rivaroxaban users from launch (October 2008) to December 2009 and encompassed potential indications for rivaroxaban use, treatment duration, and co-prescribing of potentially interacting drugs. Start of rivaroxaban treatment was defined by the date of surgery.

**Results:**

During the study period, 425 rivaroxaban users were identified contributing 440 treatment periods. For more than 82 % of these episodes labelled indications could be determined. Treatment durations exceeded recommendations in 95 % of the episodes following knee replacement whereas rivaroxaban use after elective hip surgery was found to be longer than recommended in 56 %. Prescribing of potentially interacting medication was rare except for non-steroidal anti-inflammatory drugs.

**Conclusions:**

Overall, no important off-label use of rivaroxaban was identified. Based on several assumptions that have to be considered in the interpretation of the results our study describes a database approach to reconstruct inpatient drug use for a drug started after a coded hospital procedure, when treatment continues after hospital discharge and no change in drug use is expected in the outpatient setting.

**Electronic supplementary material:**

The online version of this article (doi:10.1007/s00228-014-1697-7) contains supplementary material, which is available to authorized users.

## Introduction

Major orthopaedic surgery is associated with a high risk of venous thromboembolism (VTE), thus routine use of prophylaxis is recommended [1–3].

In Germany, post-surgical thromboprophylaxis has been traditionally conducted with low molecular weight heparins (LMWHs) or the indirect factor Xa inhibitor fondaparinux [1]. However, as these agents are administered subcutaneously, which might affect patients’ compliance, new oral anticoagulants have been developed aiming at simplifying thromboprophylaxis [4]. One of these new agents is the selective factor Xa inhibitor rivaroxaban (Xarelto®) which was approved for the prevention of VTE in adult patients undergoing elective hip or knee replacement surgery in 2008 [5]. Subsequently, approval was gained for the prevention of stroke and systemic embolism in adults with non-valvular atrial fibrillation with one or more risk factors and for the treatment of deep vein thrombosis (DVT) and pulmonary embolism (PE), and prevention of recurrent DVT and PE in 2011 and 2012 [6]. The recommended daily dose of rivaroxaban for the orthopaedic indications is 10 mg once daily for 5 weeks in patients undergoing hip replacement (HR) and for 14 days following knee replacement (KR) surgery, respectively [5, 7].

Rivaroxaban is contraindicated in patients with hepatic disease associated with coagulopathy and clinically relevant bleeding risk. Caution is to be taken in patients with severe renal impairment, and rivaroxaban use is not recommended in patients with creatinine clearance <15 ml/min. Rivaroxaban is contraindicated in pregnant or breast-feeding women and not recommended in persons up to 18 years [5, 7]. In patients receiving concomitant systemic treatment with strong inhibitors of both cytochrome P450 (CYP) 3A4 and P-glycoprotein (P-gp) use of rivaroxaban is not recommended. Additionally, strong CYP3A4 inducers should be co-administered with caution, and care is to be taken if patients are treated concomitantly with drugs affecting haemostasis [5, 7].

For new agents drug utilization studies (DUS) are increasingly required in the context of risk management plans and the evaluation of risk minimization activities e.g. exploring how medicinal products are prescribed and used in routine clinical practice and if the drugs of interest are applied within the licensed indications [8]. For this type of studies, claims databases or medical records databases are frequently used, since they are usually representative and complete for large patient populations and allow exploration of real-world utilization patterns without influencing the physicians’ prescription behaviour as it may be the case in studies using primary data collection. One drawback of these databases, however, is that drug use information usually is limited to outpatient prescriptions hampering determination of medication applied in hospital [9].

The purpose of this study was to describe how rivaroxaban was used in Germany during a time period in which approval was limited to the orthopaedic indication. This encompassed the distribution of rivaroxaban use by age, sex, potential indications, duration of use, and compliance with contraindications and precautions. This DUS also offered the opportunity to explore the feasibility of reconstructing inpatient drug use of rivaroxaban in a database where with a few exceptions inpatient prescribing information is not available.

## Methods

This retrospective cohort study was based on data from one of the four statutory health insurance providers (SHI) included in the German Pharmacoepidemiological Research Database (GePaRD). This database has been built by the Leibniz Institute for Prevention Research and Epidemiology–BIPS and contains demographic characteristics for each person, information on hospitalizations and outpatient physician visits as well as outpatient prescription data. A detailed description of GePaRD can be found in the online supplement.

The SHI providing data for this study represents a total population of about seven million insurants from all over Germany.

The study period was from October 2008 when rivaroxaban was launched in Germany to December 2009. Patients were included in the study cohort if they (i) received an outpatient prescription of rivaroxaban and (ii) had been continuously insured for at least 24 months preceding cohort entry which was defined as the reconstructed start of rivaroxaban treatment. For the licensed indications, rivaroxaban was supposed to be initiated in hospital within 6 to 10 hours after elective surgery [5, 7] and to continue after hospital discharge. An inpatient start of rivaroxaban treatment was assumed, when an outpatient prescription was observed after hospital discharge. Thus, cohort entry was defined as (i) the in-hospital date of surgery if a respective procedure could be identified via diagnostic and therapeutic procedures coded according to the Operations and Procedures Coding System (OPS), (ii) the date of hospital admission if another indication than surgery could be determined in hospital or (iii) the date of the first outpatient rivaroxaban prescription if no indication for rivaroxaban use could be identified in the hospital data. The duration of a rivaroxaban prescription was estimated by the amount of dispensed tablets which in the orthopaedic indications equalled the number of defined daily doses (DDDs). Allowing for a gap of maximum 14 days, subsequent prescriptions were considered as one treatment episode. Cohort exit was defined as the estimated end of the last rivaroxaban treatment episode, the end of the study period, death of any cause or the end of insurance, whatever happened first.

For each treatment episode, potential indications were determined in a hierarchical approach comprising four groups: (i) elective HR and KR as well as respective revisions which were approved during the study period, (ii) non-labelled orthopaedic indications such as HR or KR following fracture as well as other surgical interventions and (iii) non-labelled cardiovascular indications including atrial fibrillation and the treatment of DVT or PE. A fourth group comprised all episodes for which none of these indications could be identified. To account for the hospital stay and a possible transfer to a rehabilitation clinic, potential indications were assessed in the 90 days prior to the first outpatient rivaroxaban prescription of an episode. In two additional analyses, we extended the time window to (i) 180 days before and (ii) 90 days following this first prescription, respectively. Potential indications were identified from the in- and outpatient setting via surgical procedures and diagnoses obtained from OPS and the German modification of the International Classification of Diseases, 10th revision (ICD-10-GM) codes (codes are available on request). To differentiate between elective interventions and fracture surgery, the latter was assumed if the respective OPS codes and diagnoses indicating fracture were found during the same hospital stay. In this case, the episodes were allocated to non-labelled orthopaedic and surgical indications instead of elective KR or HR.

Presence of liver and renal impairment (ICD-10-GM codes are available on request) was assessed in the 24 months preceding cohort entry and during rivaroxaban treatment. In an additional analysis the assessment period encompassed the 180 days prior to cohort entry and the time of rivaroxaban treatment.

Based on predefined pregnancy and birth markers [10], women of childbearing age (11-50 years at cohort entry) were screened for pregnancy within 270 days prior to cohort entry up to 270 days after the estimated end of the last rivaroxaban treatment episode.

Co-medications were examined during outpatient rivaroxaban treatment and included those listed in the German Summary of Products Characteristics (SPC) [7] and were also extended to other CYP3A4 inhibitors and inducers [11].

Analyses were performed using SAS software (version 9.2; SAS Institute Inc., Cary, NC).

## Results

During the study period, 425 users of rivaroxaban met the inclusion criteria; of those, 201 were male and 224 female. Mean age at cohort entry was 63.8 years and almost two-third of them were between 60 and 79 years (Table 1). No rivaroxaban prescription was identified in patients younger than 18 years, and none of the women of childbearing age (*N* = 31) was found to be pregnant during rivaroxaban treatment. Overall, 440 continuous episodes in 425 individuals were identified with men being more likely to have multiple treatment periods than women (5.5 vs. 1.8 %). Outpatient rivaroxaban treatment was mostly initiated by general practitioners (57.0 %), followed by orthopaedic surgeons (19.3 %) and surgeons (8.6 %).

**Table 1:** Characteristics of rivaroxaban users

	Male *N* = 201	Female *N* = 224	Total *N* = 425
Age at cohort entry			
Mean (std)	64.2 (10.4)	63.4 (11.6)	63.8 (11.0)
<18 years	0 (0.0 %)	0 (0.0 %)	0 (0.0 %)
18–39 years	2 (1.0 %)	11 (4.9 %)	13 (3.1 %)
40–59 years	65 (32.3 %)	55 (24.6 %)	120 (28.2 %)
60–79 years	123 (61.2 %)	152 (67.9 %)	275 (64.7 %)
≥80 years	11 (5.5 %)	6 (2.7 %)	17 (4.0 %)
Multiple treatment episodes	11 (5.5 %)	4 (1.8 %)	15 (3.5 %)

Most of the rivaroxaban treatment episodes could be referred to HR (44.1 %) or KR (33.9 %). For 20 (4.5 %) episodes, revision of HR or KR was identified as indication (Table 2). Overall, 363 (82.5 %) episodes were found to be on-label. Non-labelled orthopaedic and surgical use including five episodes of HR or KR following fracture was determined in 8.9 %, whereas 2.5 % of treatment periods referred to non-labelled cardiovascular indications. For about 6 % of the episodes, no indication could be determined. Overall, men were more likely to receive rivaroxaban on-label than women (86.8 vs. 78.5 %).

**Table 2:** On-label and non-labelled use for rivaroxaban treatment periods stratified by sex

	Male *N* = 212	Female *N* = 228	Total *N* = 440
On-label use	184 (86.8 %)	179 (78.5 %)	363 (82.5 %)
Elective HR	107 (50.5 %)	87 (38.2 %)	194 (44.1 %)
Elective KR	68 (32.1 %)	81 (35.5 %)	149 (33.9 %)
Revision of HR	7 (3.3 %)	5 (2.2 %)	12 (2.7 %)
Revision of KR	2 (0.9 %)	6 (2.6 %)	8 (1.8 %)
Use in non-labelled orthopaedic and surgical indications	13 (6.1 %)	26 (11.4 %)	39 (8.9 %)
Use in non-labelled cardiovascular indications	3 (1.4 %)	8 (3.5 %)	11 (2.5 %)
Indication for use unknown	12 (5.7 %)	15 (6.6 %)	27 (6.1 %)

Extending the time window for the assessment of indications to 180 days before and 90 days following the first rivaroxaban prescription revealed 11 additional on-label episodes decreasing the number of treatment periods without indication to 17 (3.9 %) and increasing the proportion of on-label episodes to 85.0 %.

**Fig. 1:**
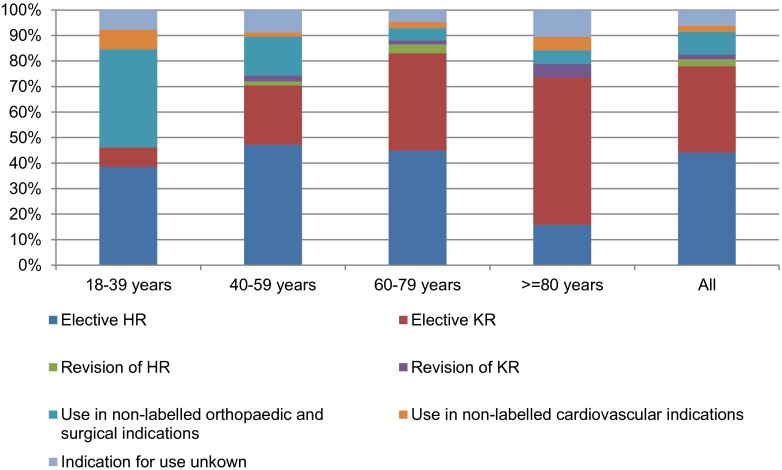
Distribution of potential indications stratified by age group

HR was the most frequent indication across almost all age groups with the exception of patients aged 80 years or older where KR was most prominent (Fig. 1). The proportion of on-label use was highest in patients aged 60 to 79 and lowest in those being 18 to 39 years old. This latter group, however, comprised only 13 patients.

Median treatment duration was 36 days after KR and 38 days following HR with the Kaplan-Meier curves showing similar shapes for both indications (see Fig. 2 in the online supplement). As displayed in Table 3, rivaroxaban treatment after elective KR was longer than the 14 days stated in the SPC in more than 95 %. These findings were less pronounced in HR where 56.2 % of the treatment duration exceeded the recommended 5 weeks. Treatment was shorter than recommended in 3.2 % of the episodes following KR and in 42.7 % after HR.

**Table 3:** Duration of rivaroxaban treatment for labelled indications

Duration of treatment episode	No. of episodes
Elective KR, revision of KR	*N* = 157
<11 days	3 (1.9 %)
11–<14 days	2 (1.3 %)
14 days	2 (1.3 %)
>14–21 days	35 (22.3 %)
>21–35 days	32 (20.4 %)
>35 days	83 (52.9 %)
Elective HR, revision of HR	*N* = 206
<4 weeks	73 (35.4 %)
4–<5 weeks	15 (7.3 %)
5 weeks	2 (1.0 %)
>5–6 weeks	58 (28.2 %)
>6 weeks	58 (28.2 %)

Recommended treatment durations for rivaroxaban according to the SPC are 14 days in patients undergoing KR and 5 weeks in those undergoing HR. The German S3-Guideline generally recommends thromboprophylaxis for 11–14 days after KR and for 4–5 weeks after HR, respectively

Regarding the 24 months preceding cohort entry and the time during rivaroxaban treatment, liver impairment was found in 65 (15.3 %) patients, whereas the definition of renal impairment was met by 24 (5.7 %). Of those patients for whom a stage of chronic renal failure could be deduced from the ICD codes (*N* = 16), none was found with a creatinine clearance <30 ml/min. The numbers of patients diagnosed with liver or renal impairment decreased to 45 (10.6 %) and 18 (4.2 %), respectively considering the shorter time window.

Frequencies of potentially interacting drugs prescribed during rivaroxaban treatment are given in Table 4. CYP3A4 inhibiting medications mainly comprised the weak inhibitor ciprofloxacin which was prescribed to 9 patients whereas prescribing of CYP3A4 inducers and P-gp inhibitors was rare. Nearly half of the patients (48.2 %) received drugs affecting haemostasis, mainly non-steroidal anti-inflammatory drugs (NSAIDs) with ibuprofen and diclofenac being used most frequently. Low-dose acetylsalicylic acid was the only platelet aggregation inhibitor prescribed. During 15 (3.4 %) rivaroxaban treatment periods, prescriptions of LMWH or fondaparinux were identified, 5 of these concomitantly on the date of the first rivaroxaban prescription. No other anticoagulants such as Vitamin K antagonists (VKA) were prescribed to patients receiving rivaroxaban.

**Table 4:** Patients receiving potentially interacting drugs prescribed in temporal relationship to rivaroxaban (rvx)

	Patients receiving potentially interacting drugs	
	During rvx treatment episodes* *N* = 440	On the day of the first rvx prescription* *N* = 440
CYP3A4 inhibitors	11 (2.5 %)	2 (0.5 %)
CYP3A4 inducers	3 (0.7 %)	2 (0.5 %)
P-gp inhibitors	6 (1.4 %)	3 (0.7 %)
Drugs affecting haemostasis	212 (48.2 %)	164 (37.3 %)
NSAIDs	203 (46.1 %)	159 (36.1 %)
Platelet aggregation inhibitors	7 (1.6 %)	1 (0.2 %)
Heparins and fondaparinux	15 (3.4 %)	5 (1.1 %)
Vitamin K antagonists	0 (0.0 %)	0 (0.0 %)

*Totals may not add up if patients received drugs from different categories

## Discussion

In this study reflecting real life practice in Germany, we examined rivaroxaban use in a cohort of more than 400 patients. We also explored an approach to investigate drug utilization for a drug which is started in hospital even though in-hospital prescribing information with a few exceptions is not available in the database providing data for this study.

This approach was based on several considerations: (i) during the study period, approval was limited to HR and KR thus a related in-hospital surgical procedure providing an exact date was likely; (ii) though thromboprophylaxis after KR or HR is recommended for only 14 days or 5 weeks, respectively, the tendency towards shorter stays in hospital after surgery [12] probably leads to a continuation of treatment in the outpatient setting where rivaroxaban use can be identified and its use extrapolated to the inpatient setting; (iii) as in Germany with hospital discharge only a very limited supply of medication e.g. to cover the weekend is given to the patient [13] an outpatient prescription within a few days after discharge could be expected; (iv) a change of treatment from subcutaneous injections of LMWH or fondaparinux in hospital to rivaroxaban after discharge was unlikely since during the time of the study rivaroxaban increased the costs for thromboprophylaxis compared to e.g. the LMWH enoxaparin or the indirect factor Xa inhibitor fondaparinux in the outpatient setting [14].

During the study period, for more than 91 % of the patients receiving an outpatient prescription of rivaroxaban, a preceding orthopaedic or surgical procedure could be identified qualifying for an in-hospital start of treatment. Overall, more than 82 % of the rivaroxaban treatment episodes were found to be on-label. Half of the remaining treatment periods were referred to other orthopaedic and surgical interventions including a small proportion of fracture surgery. Less than 3 % of the episodes were allocated to the cardiovascular indications approved in 2011 and 2012 and for 6 % no indication could be determined by our algorithm examining the 90 days preceding the first rivaroxaban prescription. However, extending the time interval to 180 days before and to 90 days following the first rivaroxaban prescription decreased the number of treatment episodes with no plausible indication to less than 4 %. Especially, including the 90 days following this first prescription yielded additional on-label indications. This might imply that rivaroxaban in these instances was prescribed prior to surgery and might partly be explained by office-based physicians with hospital affiliations conducting inpatient surgeries while prescriptions are organized by the medical practice and given to the patient before the intervention. The relatively high proportion of outpatient rivaroxaban treatment initiated by orthopaedic and other surgeons supports this assumption. Among those episodes where a potential indication for rivaroxaban use could be determined, 88 % were found to be on-label.

Overall, the recommendations on the duration of rivaroxaban use were not followed in most patients. In nearly three quarters of episodes, treatment exceeded the advised durations. The prolonged use which was mainly observed following KR might have been influenced by other guidelines such as the American College of Chest Physicians (ACCP) Evidence-Based Clinical Practice Guidelines published in 2008 (8th edition) suggesting that thromboprophylaxis for these patients be extended up to 35 days after surgery [2]. Additionally, physicians might have chosen a prolonged treatment based on a patient’s clinical condition affecting post-surgery mobilization.

On the other hand, treatment was shorter than recommended in about one quarter of episodes putting patients at potential risk of thromboembolic events. These findings mainly applied to treatment following HR. Results of a similar magnitude were also reported from the multinational Global Orthopaedic Registry (GLORY) revealing that of those who received recommended forms of VTE prophylaxis after HR or KR the duration was shorter than recommended in approximately one quarter of the patients [15].

No patients were found to be younger than 18 years at cohort entry or to be pregnant during the study period. However, in several patients, diagnoses indicating liver or renal impairment were detected, which decreased when narrowing the time window to 180 days prior cohort entry and during rivaroxaban treatment. Given the high potential of changes in liver and renal function over time, which was reflected by the two time windows examined, fewer patients may have met the criteria of impairment of liver or renal function during the short treatment period. Additionally, though GePaRD does not provide laboratory data necessary for calculation of the impairment’s severity none of those patients for whom a stage of chronic renal failure could be deduced from the ICD codes fulfilled the severity of renal impairment described as contraindications and precautions.

Though examining potentially interacting drugs beyond those stated in the SPC, prescribing of medications with possible pharmacokinetic interactions during rivaroxaban treatment was rare and included none of the drugs not recommended for concomitant use. On the contrary, nearly half of the patients received NSAIDs which may interact via pharmacodynamic mechanisms. However, these drugs constitute plausible co-medications used to reduce post-operative pain and inflammation [16]. NSAID prescriptions were slightly lower compared to a Dutch study which reported 52 % of patients receiving NSAIDs after HR or KR [17] and substantially lower than has been reported by an analysis of rivaroxaban clinical trials where over 70 % of patients with HR or KR concomitantly used NSAIDs [18]. Since these studies supposedly included also NSAIDs administered in hospital [17, 18] these differences might be referred to a higher use in the first days after surgery. Prescribing of LMWH or fondaparinux during an estimated rivaroxaban treatment may indicate a change of therapy for example because of side effects. As VTE prophylaxis has been traditionally conducted with subcutaneous injections, insufficient knowledge about the new oral anticoagulants might have contributed to these findings as well. Since no prescribing of VKA was found in temporal relationship with rivaroxaban treatment there was no hint that rivaroxaban was used as bridging therapy in patients treated with VKA before or after the surgical intervention. As this study was not designed as a safety analysis, we did not examine whether these concomitantly prescribed drugs resulted in adverse effects.

Strengths of this study are the size of the underlying population and the lack of non-response due to the nature of administrative data [19]. Determination of drug therapy based on pharmacy dispensing data is considered the gold standard as recall bias can be ruled out and information is precise in time and dose [9]. By reconstructing and including inpatient treatment, we were able to depict the complete thromboprophylaxis following orthopaedic surgery for these patients while a restriction to outpatient prescriptions would have led to an underestimation of treatment time.

Limitations are mainly attributable to the nature of the administrative data. GePaRD does not include medication bought over the counter, thus an underestimation of e.g. NSAIDs is likely. Another shortcoming of our study was that it did not include a review of individual patient files which for data protection reasons is not feasible in Germany. So, although the respective inpatient procedures could be referred to an exact date and effective VTE prophylaxis is reported to be standard care in surgical wards in Germany [20], we had no possibility to verify that in patients receiving outpatient prescriptions of rivaroxaban thromboprophylaxis was actually started in hospital on the day of surgery.

A further limitation was that the duration of rivaroxaban treatment had to be estimated based on the prescribed package sizes since GePaRD does not provide the intended length of treatment. When rivaroxaban was introduced in Germany, packages of 10 or 30 tablets were available; an additional package size of 5 tablets was marketed later. As co-payment is required per package, patients might have been prescribed larger packages and told to stop earlier which in our study would have led to an overestimation of treatment time. Based on the recommended treatment durations and the package sizes available, it is likely that this scenario applied more often to those patients receiving rivaroxaban following knee replacement.

On the other hand, an underestimation of treatment duration could have resulted from observation periods being censored by either the end of the study or patients being hospitalized leading to inpatient rivaroxaban treatment. Overall, 15 % of episodes were censored; however, this applied to only 6 % of the episodes found to be shorter than recommended.

As no direct linkage is possible between prescriptions and corresponding indications, misclassification cannot be ruled out; however, the examined hospital procedures provided detailed information allowing for a distinction between rivaroxaban use following labelled and non-labelled surgeries. By adding information from diagnoses during the same hospital stays, we were able to differentiate between elective and fracture surgery as well. Given the high proportion of on-label indications examined it seems unlikely that misclassification is of great importance.

In conclusion, our study did not identify important off-label use of rivaroxaban, apart from that of extended treatment duration which might partly result from the estimation of treatment duration based on the package size of the prescription. Additionally, given the comparatively short recommended treatment durations in both indications, our study might have missed patients who were treated according to recommendations in hospital and during a possible stay in a rehabilitation clinic without receiving any outpatient rivaroxaban prescriptions.

Based on several assumptions, our study also provides an example of reconstructing inpatient drug use in a healthcare database which does not contain prescription information in hospital when treatment is continued in the outpatient setting. This approach requires that (i) the indication of the drug of interest and thus the start of in-hospital treatment can be specifically linked to an operation or procedure code with an exact date, (ii) a change of treatment between the in- and outpatient setting is unlikely and (iii) especially for short-time treatment only a small gap between hospital discharge and the first outpatient prescription can be expected. This approach might be useful for DUS dealing with the problem of drug use starting in hospital. However, possible limitations resulting from these assumptions should be considered carefully when interpreting the results especially when estimating treatment durations.

## Electronic supplementary material

Below is the link to the electronic supplementary material.

ESM 1 (DOCX 23 kb)

Fig. 2 (DOCX 41 kb)
