# Supplementary material for: Use of rivaroxaban in Germany: a database drug utilization study of a drug started in hospital
Source: Eur J Clin Pharmacol. 2014 May 27;70(8):975–81. doi: 10.1007/s00228-014-1697-7 (PMC4088992; doi:10.1007/s00228-014-1697-7)
Supplement: Supplementary file 1 — (DOCX 23 kb) [file 228_2014_1697_MOESM1_ESM.docx]

**The German Pharmacoepidemiological Research Database (GePaRD)**

The German Pharmacoepidemiological Research Database (GePaRD) which has been built by the Leibniz Institute for Prevention Research and Epidemiology – BIPS includes data from four statutory health insurance providers (SHI). The database contains demographic characteristics for each person, information on hospitalizations and outpatient physician visits as well as outpatient prescription data.

Hospital data include information about the periods of hospitalization and the reasons for admission and discharge as well as diagnostic and therapeutic procedures coded according to the Operations and Procedures Coding System (OPS) with their respective dates. Claims of outpatient physician visits are reimbursed on a quarterly basis and contain diagnoses, treatments, and procedures. All diagnoses from the in- and outpatient setting are based on the German modification of the International Classification of Diseases, 10^th^ revision (ICD-10-GM). Prescription data are limited to reimbursable drugs and include prescribing and dispensation dates, the amount of substance prescribed and information on the prescribing physician. They are linked to a reference database providing information on the anatomical-therapeutic-chemical (ATC) code, the Defined Daily Dose (DDD), generic and trade name. With a few exceptions of mostly very expensive drugs coded by OPS codes, in-hospital medication is not included. Overall, the SHIs included in GePaRD cover more than 17 million insured members throughout Germany. Previous analyses regarding age and sex distribution, the number of hospital admissions and outpatient prescriptions have shown the database to be representative for Germany [1-3]. GePaRD has been used successfully in studies conducted in the field of anticoagulation [4-6].

References

1. Pigeot I, Ahrens W (2008) Establishment of a pharmacoepidemiological database in Germany: methodological potential, scientific value and practical limitations. Pharmacoepidemiol Drug Saf 17(3):215-223

2. Schink T, Garbe E (2010) Representativity of dispensations of non-steroidal anti-inflammatory drugs (NSAIDs) in the German Pharmacoepidemiological Research Database. Pharmacoepidemiol Drug Saf 19294

3. Schink T, Garbe E (2010) Assessment of the representativity of in-patient hospital diagnoses in the German Pharmacoepidemiological Research Database. Pharmacoepidemiol Drug Saf 19178-179

4. Behr S, Andersohn F, Garbe E (2010) Risk of intracerebral hemorrhage associated with phenprocoumon exposure: a nested case-control study in a large population-based German database. Pharmacoepidemiol Drug Saf 19(7):722-730

5. Garbe E, Kreisel SH, Behr S (2013) Risk of subarachnoid hemorrhage and early case fatality associated with outpatient antithrombotic drug use. Stroke 44(9):2422-2426

6. Jobski K, Behr S, Garbe E (2011) Drug interactions with phenprocoumon and the risk of serious haemorrhage: a nested case-control study in a large population-based German database. Eur J Clin Pharmacol 67(9):941-951
