# Supplementary material for: Use of rivaroxaban in Germany: a database drug utilization study of a drug started in hospital
Source: Eur J Clin Pharmacol. 2014 May 27;70(8):975–81. doi: 10.1007/s00228-014-1697-7 (PMC4088992; doi:10.1007/s00228-014-1697-7)
Supplement: Supplementary file 2 — (DOCX 41 kb) [file 228_2014_1697_MOESM2_ESM.docx]

**Kaplan-Meier curve for patients treated with rivaroxaban following elective hip or knee replacement**


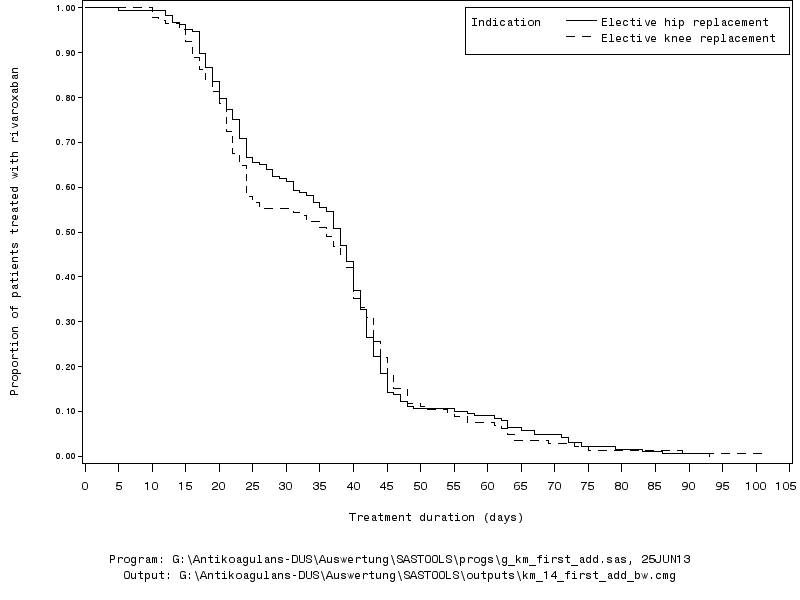


Figure 2: Kaplan-Meier curve for patients treated with rivaroxaban following elective hip or knee replacement
